# Actions on social determinants and interventions in primary health to improve mother and child health and health equity in Morocco

**DOI:** 10.1186/s12939-016-0309-9

**Published:** 2016-02-02

**Authors:** Wiam Boutayeb, Mohamed Lamlili, Abdellatif Maamri, Souad Ben El Mostafa, Abdesslam Boutayeb

**Affiliations:** URAC04, LaMSD, Department of Mathematics and Informatics, Faculty of Sciences, University Mohamed Ist, Oujda, Morocco; ISPITS Oujda, Health Ministry, Oujda, Morocco; ISPITS Oujda Annex Nador, Health Ministry, Oujda, Morocco

**Keywords:** Interventions, Primary health, Social determinants, Health equity, Maternal mortality, Infant mortality

## Abstract

**Background:**

Over the last two decades, Moroccan authorities launched a number of actions and strategies to enhance access to health services and improve health outcomes for the whole population in general and for mother and child in particular. The Ministry of Health launched the action plans 2008–2012 and 2012–2016 and created the maternal mortality surveillance system. The Moroccan government opted for national health coverage through a mandatory health insurance and a scheme of health assistance to the poorest households. Other initiatives were devoted indirectly to health by acting on social determinants of health and poverty reduction. In this paper, we present results of an evaluation of interventions and programmes and their impact on health inequity in Morocco.

**Method:**

We used data provided by national surveys over the last decades, information released on the website of the Ministry of Health, documentation published by the Moroccan government and international reports and studies related to Morocco and published by international bodies like the World Health Organisation, United Nations Development Programme, United Nations Population Fund, UNICEF, UNESCO and the World Bank.

A short review of scientific publications was also carried out in order to select papers published on health equity, social determinants, health system and interventions in primary health in Morocco.

Inferential and descriptive statistics (including principal component analysis) were carried out using software SPSS version 18.

**Results:**

The findings indicate that substantial achievements were obtained in terms of access to health care and health outcomes for the whole Moroccan population in general and for mothers and children in particular. However, achievements are unfairly distributed between advantaged and less advantaged regions, literate and illiterate women, rural and urban areas, and rich and poor segments of the Moroccan population.

**Discussion:**

Studies have shown that it is difficult to trace the effect of a primary health intervention on the access to health care due to synergetic and overlapping effect of interventions and initiatives aiming to improve the wellbeing of the Moroccan population. Descriptive and inferential statistics were used to illustrate the correlation existing between different variables measuring access to health and health outcomes on one side and variables like income, education, employment and health staff on the other side.

**Conclusion:**

In Morocco, average access to health care and services as well as health outcomes have improved during the last decades. However, socio-economic inequalities and health inequity are persistent. The present study indicates that urgent and efficient actions on social determinants of health are needed in order to sustain average achievements and improve health equity for the whole Moroccan population.

## Background

Over the last two decades, Moroccan authorities launched a number of actions and strategies to enhance access to health services and improve health outcomes for the whole population in general and for mother and child in particular. The relatively uncomfortable human development rank of Morocco and the engagement to achieve the Millennium Development Goals lead the Moroccan government to develop a national strategy to reduce maternal and neonatal mortality. The Ministry of Health launched the action plans 2008–2012 [1] and 2012–2016 [2] and created the maternal mortality surveillance system [3]. The low health insurance coverage (16 %) lead to the mandatory health insurance (Assurance Maladie Obligatoire) for public and private sector employees, a scheme of health assistance to the poorest households (Régime d’Assistance Médicale RAMED) [4] and Medical Insurance for Independents. Other initiatives were devoted indirectly to health by acting on social determinants of health and poverty reduction. The commitment was made at the highest level by King Mohamed VI who launched the education charter in 2000, the study of reflection and debate to evaluate “50 years of human development in Morocco” in 2003, the Equity and Reconciliation Commission and the Moroccan Family code (Moudawana) in 2004, and the National Initiative Human Development in 2005. Equality in access to health care and services and equity in spatial distribution of health resources were clearly indicated in the new constitution adopted in 2011.

In this paper, we present results of an evaluation of interventions and programmes by analysing simultaneously the evolution of access to health care and health outcomes especially for mothers and children. As the quasi-totality of indicators are averages that may hide large inequalities in access to health as well as in health outcomes, we also consider the impact of strategies and action on health inequity in Morocco.

## Method

We used data provided by national surveys over the last decades, including: The national population and family health survey (ENPS 1992) [5], The Survey on Health and Health System Reactivity (ESRSSM 2003) [6], The High Planning Commission National Survey on Household Consumption and Expenditure (ENCDM, 2000/01) [7], The national population and family health survey (ENPSF 2003/04) [8], The High Planning Commission National Survey on Income and Household level of Life (ENRNVM, 2006/2007) [9], The National Survey with Multiple Indicators and Youth Health (MICS3 2006–2007) [10], The High Planning Commission National Demographic Survey (ENDPR 2009–2010) [11], The national population and family health survey (ENPSF 2011) [12], The High Planning Commission Millennium Development Report 2012 [13], The National Observatory Human Development Household Panel Survey 2012 [14]. We also considered data released on the website of the Ministry of Health (healthy figures, annual reports) [15], documentation published by the Moroccan government and international reports and studies related to Morocco and published by international bodies like the World Health Organisation (WHO) [16, 17], UNDP [18], UNFPA [19], UNICEF [20], UNESCO [21] and the World Bank [22]. Finally, a short review of scientific publications was carried out in order to select papers published on health equity, social determinants, the Moroccan health system and interventions in primary health in Morocco [23–33].

We used descriptive and inferential statistics (principally chi-square tests with (*p* < 0.05) as level of significance) to analyse data and compare evolution of different health and socio-economic indicators according to social determinants of health like income-consumption, education, milieu of residence and regions. Principal component analysis was also used for multidimensional illustration of similarities and dissimilarities between 13 geographic regions of Morocco based on correlations between 12 variables.

## Results

### Actions on social determinants of health and interventions in primary health care

By the end of the second millennium, three programmes were devoted to rural areas, namely: programme providing drinking water, programme of electrification in rural globally and national programme of rural roads. Consequently, access to the drinking water network increased from 92.4 % in 1999 to 96.5 % in 2011 in urban areas and significantly from 15.5 to 48.8 % in rural areas during the same period (*p* < 0.05). Similarly, access to electricity increased from 89.3 % in 1999 to 98.5 % in 2011 in urban zones and from 23.2 to 88.2 % in rural zones (*p* < 0.05). With 89.3 % in urban areas and only 5 % in rural areas, access to sanitation is still a challenge (*p* < 0.01). Programmes of struggle against waterborne diseases are amongst ongoing health programmes.

The third Millennium provided a golden opportunity for Moroccan authorities to evaluate a half century of independence (obtained in 1955) and to analyse the state of art of all sectors in general and that of health in particular. In 2003, the King of Morocco called for a study of reflection and debate to evaluate “50 years of human development in Morocco”. The report released in 2004 showed that, though a substantial improvement has been achieved in the average living standard of the whole population. Morocco remained a contrasting country with large disparities between regions, an unacceptable gap between rich and poor, and a rural world lagging far behind the urban cities [22, 25–29]. Following this and aiming to reduce poverty and health inequity, the King of Morocco launched the National Initiative for Human Development in 2005 with the following priorities: 1) to struggle against poverty in rural areas, 2) to combat social exclusion in urban areas and 3) to fight against vulnerability.

A programme to reduce illiteracy allowed to increase the proportion of literate Moroccan aged 10 years and plus from 45.3 % in 1994 to 55.5 % in 2012 [14]. However, gender inequality and milieu-region disparity show that in 1994, rural women (10.9 %) were seven times less likely to be literate than urban males (75.3 %) (*p* < 0.05). The gap was reduced to 2.2 (84.1/38.7) in 2012.

Similarly, according to wealth, rural women from the poorest wealth quintile (36.1 %) are 2.6 times less likely to be literate than urban males from the richest quintile (92.7 %) (*p* < 0.05). Consequently, one can conclude that a rural poor woman suffers from three handicaps compared to an urban rich male.

The High Commission for Planning surveys (ENCDM 2000/2001 and ENRNVM 2006/2007) [7, 9] and the Observatory Human Development Household survey (EPM 2012) [14] allow a comparison between the levels of expenditure in rural an urban areas and also according to wealth quintiles. With a yearly increase of 12 %, the annual expenditure per capita has more than doubled in ten years, between 2001 (8280 MAD) and 2012 (19267 MAD) but with nearly the same urban–rural ratio of 2.1 (10642/5288) in 2001 and 2.13 (23687/11101) in 2012. Over the same period, while expenditure of the poorest 10 % decreased from 2.6 to 2.1 %, the richest 10 % of the Moroccan population increased their expenditure from 32.1 to 36.8 %, showing that the ratio richest 10 %/poorest 10 % increased from 12.2 to 16.2 (Table 1).

**Table 1:** Evolution of expenditure per capita (value in MAD and % of total expenditure) [7, 9, 14]

Year	National	Urban	Rural	Urban	D1	D10	Ratio U/R	Ratio D10/D1
2000/2001	8280	10642 (67 %)	5288 (33 %)	10642 (67 %)	2179 (2.6 %)	26615 (32.1 %)	2.01	12.2
2006/2007	11233	13894 (64 %)	7777 (36 %)	13894 (64 %)	2960 (2.6 %)	37175 (33.1 %)	1.79	12.6
2012	19267	23687 (68 %)	11101 (32 %)	23687 (68 %)	4261 (2.1 %)	69021 (36.8 %)	2.13	16.2

The evolution of income per capita is similar to the evolution of expenditure per capita.

**Fig. 1:**
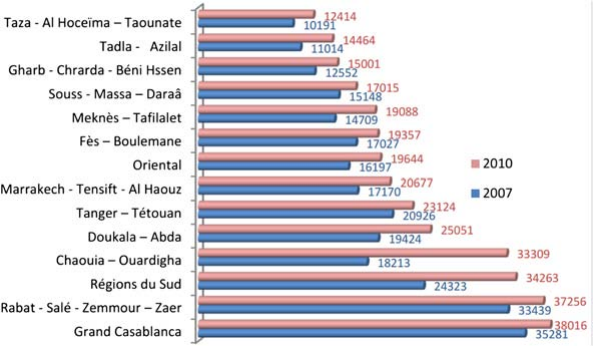
Evolution of GDP per capita (MAD) by region [34]

Figure 1 below shows regional disparities in income per capita, indicating that, the average income per person living in the most advantaged region (38016 MAD) is more than 3 times higher than the average income per person living in the least advantaged region (12414 MAD).

**Fig. 2:**
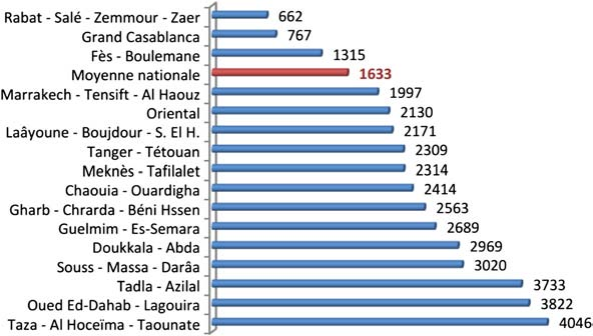
Inhabitant per physician by region in 2011 [34]

The number of basic health care facilities increased by 63 % between 1990 (1653) and 2011 (2689) (from one facility for 14600 inhabitants to one for 12000 inhabitants). The number of public hospitals increased from 52 in 1960 to 142 in 2011, of which 40 are specialised hospitals. It should also be indicated that health care improved with the creation of three hospital universities in Fez, Marrakech and Oujda during the last decade and should be more enhanced by the ongoing building process of two new hospital universities in Agadir and Tangier. For nurses and midwives, twenty three Higher Institutes of Nurses and Health Techniques were recently created or transformed to the standardized LMD system under higher education ministry. The number of doctors in public and private sector increased regularly at a rate of 9 % between 1992 and 2012 but the number of inhabitants per physician is unfairly distributed by region as indicated in Fig. 2. Finally, prices of 1600 drugs were reduced in 2014 and another list of 98 drugs followed in 2015.

With just 15 % of national health insurance coverage by the end of the second millennium, more than 33 % of the population was unable to afford necessary medical care and out of pocket represented 60 % of the total health expenditure. To overcome the huge shortage in health insurance and inequity (Table 2), the government opted for a mandatory health insurance for public and private employees which started in 2005, a scheme assistance for the poor which was implemented at the national level in 2011 and student insurance which has just been approved by the parliament in May 2015. According to the Ministry of Health, health insurance coverage reached 60 % of the Moroccan population in May 2015 and should reach 92 % by the ongoing implementation of medical insurance for independents expected to cover 11,000,000 people and 288000 students.

**Table 2:** Percentage of population with health insurance coverage [14]

	Q1		Q2		Q3		Q4		Q5		Total	
	2006	2012	2006	2012	2006	2012	2006	2012	2006	2012	2006	2012
Urban	4.9	16.7	8.4	26.8	18.8	34.0	27.7	44.5	44.3	60.2	25	33.4
Rural	1.0	4.2	1.1	5.8	4.9	8.5	8.1	8.6	12.5	14.7	3.8	7.6
National	2.2	7.3	4.6	15.7	13.0	22.4	20.8	33.0	38.5	52.9	15.8	23.3

By the dawn of the third Millennium, around 60 % of deliveries were occurring in public and private health services while the remaining 40 % occurred at home. The maternal mortality ratio was 227 for 100, 000 live births, and the neonatal mortality rate was 27 for 1000 live births. The national population and family health survey (ENPSF 2003/04) revealed that the main obstacles to access to emergency obstetric care were financial barriers (for 74 % of women), distance to a health facility (60 %) and transport (46 %). In 2007, maternal and neonatal mortality became a priority issue in Morocco and lead the Ministry of Health to adopt the Maternal Mortality Strategy illustrated by the action plan 2008–2012 which aimed to reduce Maternal Mortality Rate (MMR) from 227 to 50 deaths for 100,000 live births, by: 1) reducing barriers to access to emergency obstetric services, 2) enhancing the quality of health care and 3) improving governance [1]. The Ministry of Health also launched the maternal mortality surveillance system which allowed for analysis of data collected in 2009 and revealed that the goal of MMR = 50 was not achievable by 2015. A new action plan 2012–2016 was launched in order to consolidate the results achieved, reinforce proximity management and target efficient actions for rural areas and disadvantaged regions.

### Access to health services and health care

Access to health services and health care has risen significantly during the last decades as shown in Table 3. Indeed, the percentage of deliveries assisted by skilled personnel increased from 31 % in 1992 to 73.5 % in 2011. Similarly, the percentage of women having at least one prenatal visit increased significantly from 33 to 77.1 % during the same period (*p* < 0.05). The postnatal care also increased but it remains at a low level (21.9 %). The contraception prevalence and vaccination of infant aged 12–23 months increased significantly from 41.5 and 75.7 % in 1992 to 67.4 and 99.6 % in 2011, respectively (*p* < 0.05). The percentage of caesarean sections increased from 2 % in 1992 to 9.6 % in 2011.

**Table 3:** Evolution of access to health care [5, 8, 12]

Health service or health care		1992	2003-2004	2011
% of deliveries assisted by medical skilled personnel	Urban	64	85	92.1
	Rural	14	40	55
	National	31	63	73.5
% of women having at least one prenatal visit	Urban	61	85	91.6
	Rural	18	48	62.7
	National l	33	68	77.1
% of women having postnatal visits	Urban	n.a	16.3	30.5
	Rural	n.a	3.6	13.3
	National	n.a	6.6	21.9
% of women aged 15–49 years using any kind of contraceptive method	Urban	54.4	65.5	68.9
	Rural	31.6	59.7	65.5
	National	41.5	63	67.4
% of vaccination coverage for infant aged 12–23 months	Urban	93.7	93.5	99.8
	Rural	66.7	86.8	100
	National	75.7	89.1	99.6
% of caesarean sections	National	1.9	5.6	9.6

### Health outcomes

**Fig. 3:**
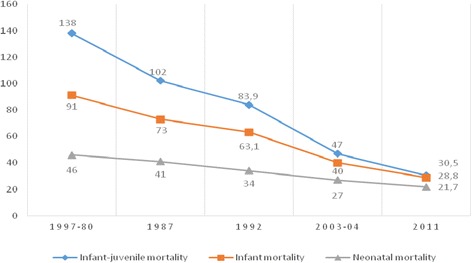
Neonatal, Infant and Under-five mortality rates (per 1000 live births) [5, 8, 12, 13]

As indicted by Fig. 3 below, neonatal, infant and under-five mortality have significantly decreased over the last 3 decades. Morocco seems on the track to achieve the fourth Millennium Development Goal in terms of infant mortality and under-five mortality while the neonatal mortality goal remains out of reach. Indeed, infant mortality decreased significantly from 63.1 deaths for 1000 live births in 1992 to 28.8 in 2011 (*p* < 0.05). Over the same period, the under-five mortality rate has seen a more accelerated decrease from 83.9 to 30.5. In opposition, the neonatal mortality decreased from 34 to 21.7 [5, 8, 12, 13].

The maternal mortality decreased from 227 in 2000 to 112 in 2010 [19]. Life expectancy at birth increased by nearly 16 years during the last 3 decades, from 59.1 years in 1980 to 74.8 years in 2010. However, there is a gap of nearly 6 years between urban population (77.3 years) and rural population (71.7 years).

The proportion of children suffering from stunting decreased from 22.6 % in 1992 to 14.9 % in 2011. Deficiency in Iron, vitamin A and vitamin D affect respectively one third of children aged 6 months to 5 years, 20 and 10 % of under 5 years children. Similarly, pregnant women have an anaemia prevalence of 37.2 %.

### Health equity

Despite the important achievements in terms of access to health care and health outcomes and endeavours to reduce inequalities, health equity remains a challenge for Moroccan health decision makers. The country is still facing regional disparities, urban–rural differences, education gradient and the wealth-health gaps in access to health care and health outcomes.

**Fig. 4:**
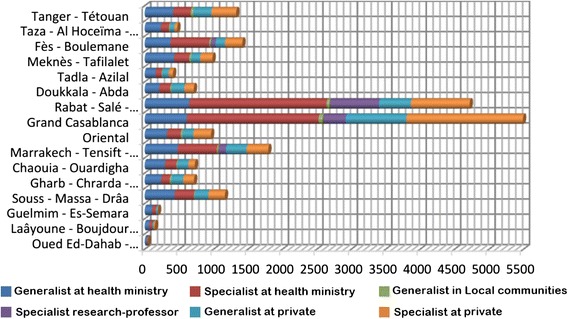
Doctors employer organization and the sector by region in 2011 [34]

The total number of doctors increased from 3779 in 1992 to 11812 in 2012 in the public sector and from 3324 to 7934 in the private sector, during the same period. However, the regional distribution is very inequitable as indicated by Fig. 4 [34].

As indicated in Table 4, assisted deliveries, prenatal care and postnatal visits vary considerably with milieu, education level and wealth quintile. The last survey (2011) shows that the proportion of deliveries assisted by skilled personnel in urban areas (92.1 %) is nearly the double of that occurring in rural areas (55 %). Similarly, women with a primary certificate (99 %) are more likely to be assisted by skilled personnel than women without certificate (66 %). The probability of delivery assisted by skilled personnel is 2.5 higher in the richest quintile (96 %) compared to the poorest quintile (37.7 %). Prenatal visits with at least one visit follow nearly the same pattern of inequalities than the assisted deliveries. However, prenatal visits as suggested by WHO (at least four visits) show more exacerbated differences, reaching a more than 5 fold advantage in the richest quintile (71.7 %) compared to the poorest quintile (13.8 %) and a ratio of 2.5 between urban (60 %) and rural (25 %), and between mother with primary certificate (84 %) and mother without certificate (34 %).

**Table 4:** Inequality in access to health care [5, 8, 12]

	Assisted deliveries by skilled personnel			Prenatal visits (one visit or plus)			Prenatal visits (Four visits or plus)
	1992	2004/03	2011	1992	2004/03	2011	2011
Urban	63.7	85.3	92.1	60.6	85	91.5	60
Rural	13.8	39.5	55.0	17.6	48	62.7	25
Mother without education	20.6	48.8	66.0^a^	23.2	55.5	70.3^a^	34^a^
Mother with secondary level or +	91.3	94.4	99.0^a^	87.4	93.4	98.9^a^	84^a^
Poorest quintile (Q1)	na	29.5	37.7	na	39.7	49.6	13.8
Second quintile (Q2)	na	49.4	66.6	na	56.5	69.3	29.8
Third quintile (Q3)	na	70.3	86.6	na	70.6	85.2	48.6
Fourth quintile (Q4)	na	86.1	91.1	na	86.8	93	60.3
Richest quintile (Q5)	na	95.4	96.0	na	93.1	97.3	71.7
National	31	62.6	73.6	32.1	67.8	77.1	42.6

^a^ In 2011: mother with (primary) certificate vs mother without certificate

**Fig. 5:**
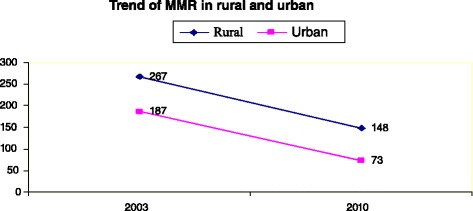
Trend of MMR in urban and rural [19]

For maternal mortality, available data don’t allow a comparison between educated and non educated women nor between rich and poor women. However, the maternal mortality ratio in rural areas (148) is more than double of that in urban areas (73) (Fig. 5).

At the national level, neonatal mortality decreased from 34 in 1992 to 21.7 in 2011 but the ratio rural/urban increased from 1.2 to 1.5 during the same period. Infant mortality and under five mortality have seen reduction in gaps between rural and urban areas, richest and poorest quintiles, and level of education. However, inequity is still there and more attention should be given to the least advantaged households (Table 5).

**Table 5:** Evolution of infant mortality [5, 8, 12]

	NNM			IMR			U5MR		
	1992	2004	2011	1992	2004	2011	1992	2004	2011
Rural	29.9	33	24.7	69.3	55	33.5	97.8	69	35
Urban	36.2	24	18.3	51.9	33	23.6	58.7	38	25.4
Ratio rural/urban	1.21	1.37	1.35	1.33	1.67	1.42	1.67	1.81	1.38
Mother without education	35.9	33	24*	67.7	52	31.8*	91.3	63	33.3*
Mother with secondary level or plus	13.8	17	15.5*	20.9	23	20.5*	22.4	27	22.6*
Ratio without education/secondary level or plus	2.53	1.94	1.55	3.24	2.26	1.55	4.07	2.33	1.47
The poorest quintile (Q1)	na	38	24.9	na	62	33.9	na	78	35.6
The medium quintile (Q3)	na	25	25.1	na	37	33.6	na	47	34
The richest quintile (Q5)	na	19	15.1	na	24	18.7	na	26	21.1
Ratio Q5/Q1		2	1.65		2.58	1.81	na	3	1.61
National	34	27	21.7	63.1	40	28.8	83.9	47	30.5

*In 2011: mother with (primary) certificate vs mother without certificate

na: non available

The national proportion of children suffering from stunting decreased from 22.6 % in 1992 to 14.9 % in 2011 but the ratios rural/urban and poorest quintile/richest quintile increased during the same period, showing that in 2011, more than 20 % of rural children and nearly 30 % of poor children suffer from stunting (Table 6).

**Table 6:** Proportion of under-five children suffering from stunting [5, 8, 12]

	1992	2003/2004	2011
National	22.6	18.1	14.9
Rural	27.7	23.6	20.5
Urban	13.1	12.9	8.6
Ratio rural/urban		1.83	2.38
Mother without education	25.3	21.8	na
Mother with secondary level or plus	6.1	10.5	na
Ratio without education/secondary level or+		2.08	na
Developed Region (DR)	na	28.2	24.4
Less developed Region (LDR)	na	8.7	5.1
Rapport DR/LDR		3.24	4.78
Poorest Quintile (Q1)	na	29.1	28.3
Medium Quintile (Q3)	na	16.1	10.3
Richest Quintile (Q5)	na	10.2	6.7
Ratio Q5/Q1		2.85	4.22

In 2011: mother with (primary) certificate vs mother without certificate

## Discussion

**Fig. 6:**
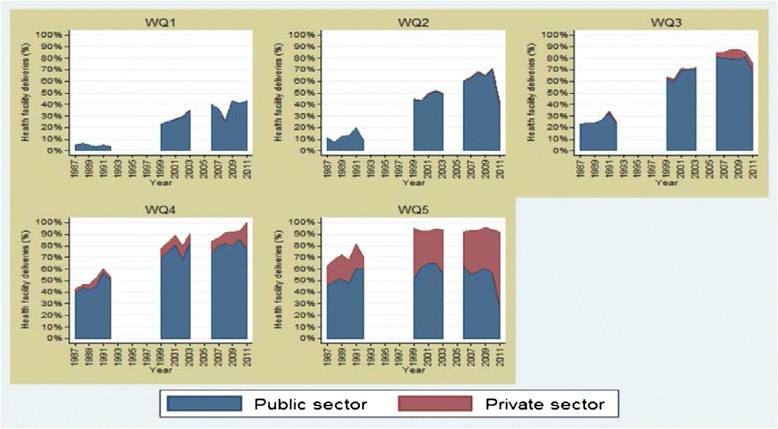
Trend in deliveries taking place in public and private sector facilities stratified by relative wealth, in Morocco, 1987–2011 (DHS data) (Reproduced with author’s permission) [32]

Over the last 3 decades, Morocco has substantially improved access to health services and health care to all Moroccans in general and to mothers and children in particular. Life expectancy increased, maternal and mortality decreased and health outcomes in general have improved. It is, however, difficult to trace the effect of a primary health intervention on the access to health care due to synergetic and overlapping effect of interventions and initiatives aiming to improve the wellbeing of population by acting on social determinants of health like education, health coverage, poverty reduction, drinking water, activities generating of income, etc.…. For example, studies have found that, in Morocco, the increase in facility-based delivery and caesarean sections was multi-factorial. The “fee exemption for maternal health care” (FEMHealth) project carried out comprehensive evaluations of the impact, cost and effectiveness of the removal of user fees for delivery care on maternal and neonatal health outcomes and service quality in West Africa and Morocco [32, 33]. For Morocco, the FEMHealth report found no evidence of a change post-2008 (date of launch of free access to emergency obstetric care) in facilities deliveries and stressed that inequality reduction may be explained by the fact that richer women already had high coverage (Fig. 6).

**Fig. 7:**
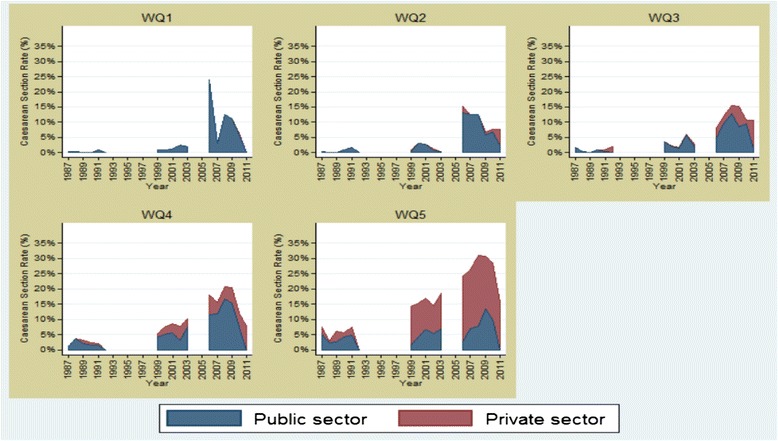
Trend in caesarean sections taking place in public and private sector facilities stratified by relative wealth, in Morocco, 1987–2011 (DHS data) (Reproduced with author’s permission [32]

The FEMHealth report also indicates that the top quintile makes substantial and growing use of the private sector deliveries while the middle poorer groups are actually closer to the WHO’s optimal rate of caesarean (10–15 %), concluding that “Morocco caesarean section rate is at a level where any increase would be unlikely to lead to further reductions in maternal mortality, and if anything the concern is excess and unnecessary caesareans among certain groups” (Fig. 7) [32].

A similar study was carried out by Cresswell et al. who examined trends in the utilisation of facility-based delivery and caesareans between 1987 and 2012 in order to see whether uptake of facility deliveries and caesareans accelerated at the time of major changes in policy, including the periods: 1994 (the family planning/maternal child health phase V project), 2005–2006 (introduction of health insurance programmes) and 2008 (the action plan to reduce maternal mortality). They concluded that “success in improving uptake facility deliveries and caesarean is likely to be the result of the synergistic effects of comprehensive demand and supply-side strategies, including a major investment in human resources and free delivery care”. They found no evidence of acceleration in trend coinciding with any of the policies or programmes investigated. Each programme was complementary [31].

The achievements in terms of access to health care and health outcomes remain challenged by unacceptable disparities and inequalities. Moreover, health equity in Morocco should be considered both as a quantitative and qualitative issue as illustrated by the following studies [23, 24, 31, 33].

The National Expert Committee which analyzed the data collected by the Maternal Mortality Surveillance System from the 1^st^ January 2009 to 31^st^ December 2009 revealed striking findings. Indeed, it was indicated that 71.6 % of maternal deaths occurred in public hospitals, 23.8 % of deaths occurred at home or during transit while no death occurred in private hospitals [3]. The paper published by Abouchadi et al. on “Preventable maternal mortality in Morocco: the role of hospitals”, raised a crucial point by indicating that “simply reaching the hospital may not be sufficient to manage obstetric complications and save mothers’ live” [23]. The authors reported that “almost 80 % of the maternal deaths that occurred in Morocco in 2009 were avoidable and that in more than half of the hospital deaths substandard care in hospital was involved”. It was stressed that such a high proportion of avoidable deaths was shocking for the National Expert Committee members, policymakers and practitioners. These figures suggest that health outcomes depend not solely on the rate of access to health services but also on the quality of health care provided. They consequently suggest a qualitative focus in order to understand why these avoidable deaths occurred and how they were related to social determinants like socioeconomic status and education of women on the one hand, and substandard care in hospitals, competence and motivation of health personnel on the other hand [33].

An interesting study by Assarag et al. on the of severe maternal morbidity referred to as “near misses” revealed that low education level, lack of antenatal care, presence of obstetric complications during pregnancy, labor duration >24 h before reaching a healthcare facility, and time to reach the final place of care >1 h were the main determinants of near misses. They found that women who did not receive antenatal care were 8 times more likely to be near-miss cases and that the main reasons given by the women for the first delay (time at home before visiting a health facility) were lack of a parent’s authority to go to a facility and fear of the health facility [24].

Spatial disparity is one of the main social determinants of health in Morocco. In order to get an overview of the situation, principal component analysis (PCA) is used with 13 Moroccan regions (for which data was available) as individuals and a set of 12 variables (antenatal care (4 visits or +)%, delivery assistance%, contraception%, vaccination%, employed women%, per capita GDP, Children with diarrhea%, children with suspected pneumonia%, stunting children%, young illiteracy%, under five mortality rate (U5MR), need in medical staff%). The aim of PCA is to summarise the information and get an easy comparison between the 13 regions after projection of variables and individuals on the first plan given by factors F1 and F2. The corresponding eigenvalues give the percent of information explained by these factors respectively. In our case, the first and second axes explain 55.7 and 15.% respectively. Consequently, we have a representation on the first plan (F1 × F2) which summarises more than 70 % of information. Additional information may be obtained by the third and fourth axis, but the interpretation is then conditional to what was given by the precedent factors.

**Fig. 8:**
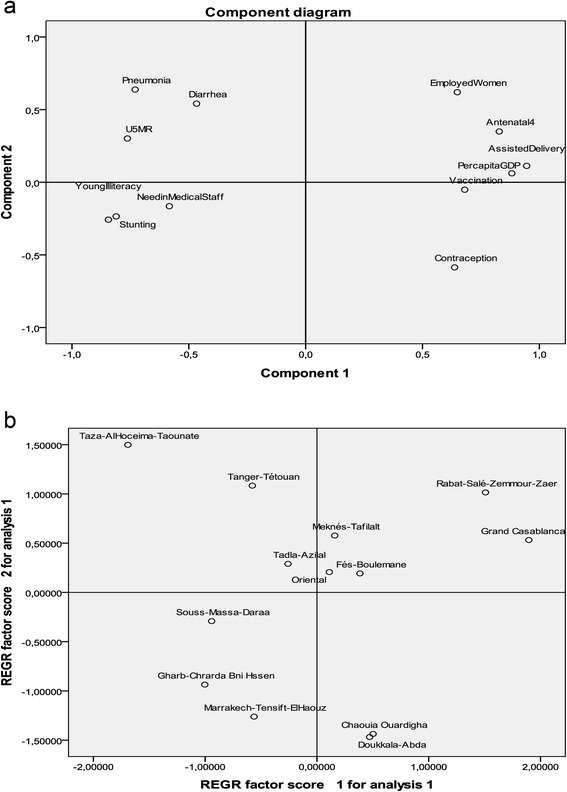
**a** Projection of the 12 variables on the first plane (F1xF2) given by PCA, **b** Projection of the 13 regions on the first plane (F1xF2) given by PCA

We get an interesting sketch by projecting the 12 variables and the 13 individuals on the first plane (F1 × F2). Figure 8 (a) shows that the first axis (F1) is mainly determined by two sets of variables. On the right hand side of the plane, beside per capita GDP and percent of employed women, we find variables representing access to health care and services like vaccination, contraception, delivery assistance and antenatal care. The left hand side of the plane shows a second group of variables including, under five mortality rate, stunting, diarrhea, pneumonia, young illiteracy and need in medical staff. The second axis (F2) is mainly determined by the variables employed women, diarrhea and pneumonia opposed to contraception. More details on the corelation between different variables is provided by the matrix correlation, showing for instance, that young illiteracy is negatively correlated with antenatal care (−0.914) and assisted delivery (−0.858) while under five mortality is positively correlated with pneumonia (0.761) and stunting (0.542) (Table 7).

**Table 7:** Correlation matrix provided between the 12 variables used in PCA

	Employed Women	Contraception	Antenatal 4+	Assisted Delivery	Stunting	Diarrhea	Vaccination	Pneumonia	Young Illiteracy	Need in Medical Staff	U5MR	Per capita GDP
Employed Women	1	0,051	0,708	0,624	−0,681	−0,077	0,237	−0,097	−0,613	−0,525	−0,38	0,624
Contraception	0,051	1	0,321	0,65	−0,401	−0,477	0,414	−0,792	−0,376	−0,228	−0,547	0,498
Antenatal 4+	0,708	0,321	1	0,871	−0,626	−0,26	0,523	−0,441	−0,941	−0,356	−0,366	0,726
Assisted Delivery	0,624	0,65	0,871	1	−0,806	−0,351	0,604	−0,594	−0,858	−0,549	−0,562	0,802
Stunting	−0,681	−0,401	−0,626	−0,806	1	0,379	−0,449	0,35	0,613	0,66	0,542	−0,661
Diarrhea	−0,077	−0,477	−0,26	−0,351	0,379	1	−0,103	0,678	0,219	0,321	0,374	−0,257
Vaccination	0,237	0,414	0,523	0,604	−0,449	−0,103	1	−0,531	−0,641	−0,414	−0,566	0,498
Pneumonia	−0,097	−0,792	−0,441	−0,594	0,35	0,678	−0,531	1	0,501	0,253	0,761	−0,619
Young Illiteracy	−0,613	−0,376	−0,941	−0,858	0,613	0,219	−0,641	0,501	1	0,245	0,458	−0,773
Need in Medical Staff	−0,525	−0,228	−0,356	−0,549	0,66	0,321	−0,414	0,253	0,245	1	0,432	−0,377
U5MR	−0,38	−0,547	−0,366	−0,562	0,542	0,374	−0,566	0,761	0,458	0,432	1	−0,815
Per capita GDP	0,624	0,498	0,726	0,802	−0,661	−0,257	0,498	−0,619	−0,773	−0,377	−0,815	1

Figure 8 (b) summarises the effect of variables on individuals and consequently, yields a spatial disparity with similarities and dissimilarities between the 13 Moroccan regions. The first axis (horizontal axis) may be interpreted as a summary indicator of advantage in terms of all variables and especially health accessibilty and health outcomes. For instance, the region Taza-AlHoceima-Taounate appears isolated on the left hand right side of the plane due the fact that this region has the lowest per capita GDP and nearly the lowest value of all variables of access to health (delivery assistance, antenatal care and vaccination), the maximum values of diarrhea and pnumonia, and a very high rate of U5MR, stunting, young illiteracy and need of medical staff. In opposition, we find the two advantaged regions of Grand Casablanca and Rabat-Salé-Zemmour-Zaer with the highest per capita GDP and rate of employed women, high values of delivery assistance, antenatal care and vaccination combined with the lowest values of U5MR, need of medical staff, young illiteracy, pneomonia and stunting.

Similar comparisons can be made on the second axis (F2 vertical). For instance, while Tanger-Tetouan and Marrakech-Tensift-Alhaouz have nearly the same projection on the first axis (horizontal), their projections on the second axis (vertical) are nearly opposed, mainly due to the effect of variables contraception, employed women and pneomonia. Indeed, Tanger-Tetouan has the lowest value of contraception (60.2 %) and high values of pneumonia (12.5 %) and employed women (17.6 %) whereas Marrakech-Tensift-Alhaouz has the highest value of contraception (72.5 %) and relatively low values of pneomonia (7.1 %) and employed women (8.3 %).

## Conclusion

Although access to health care and services as well as health outcomes improved in average during the last decades, socio-economic inequalities and health inequity are persistent according to milieu of residence (rural–urban), wealth status, level of education and spatial disparity between different geographic regions. The correlation between variables measuring access to health and health outcomes on one side and variables like income, education, employment and health staff on the other side indicates that urgent and efficient actions on social determinants of health are needed in order to sustain average achievements and improve health equity for the whole Moroccan population.
